# Voriconazole-Induced Periostitis Mimicking Chronic Graft-versus-Host Disease after Allogeneic Stem Cell Transplantation

**DOI:** 10.1155/2016/3242196

**Published:** 2016-06-14

**Authors:** Karen Sweiss, Annie Oh, Damiano Rondelli, Pritesh Patel

**Affiliations:** ^1^Department of Pharmacy Practice, University of Illinois at Chicago, Chicago, IL 60612, USA; ^2^Cancer Center, University of Illinois, Chicago, IL 60612, USA; ^3^Division of Hematology/Oncology, University of Illinois at Chicago, Chicago, IL 60612, USA

## Abstract

Voriconazole is an established first-line agent for treatment of invasive fungal infections in patients undergoing allogeneic stem cell transplantation (ASCT). It is associated with the uncommon complication of periostitis. We report this complication in a 58-year-old female undergoing HSCT. She was treated with corticosteroids with minimal improvement. The symptoms related to periostitis can mimic chronic graft-versus-host disease in patients undergoing HSCT and clinicians should differentiate this from other diagnoses and promptly discontinue therapy.

## 1. Introduction

Invasive fungal infections lead to significant morbidity and mortality in patients who are undergoing allogeneic stem cell transplantation (ASCT) [1–3]. Treatment is typically prolonged and continues until complete clinical and radiographic resolution. Voriconazole is an established first-line treatment for invasive aspergillosis [2]. Although mostly well tolerated, it is associated with the uncommon yet clinically relevant complication of periostitis [1]. Here we report this underrecognized complication of voriconazole and its unique implications in the setting of ASCT.

## 2. Case Report

A 58-year-old woman with intermediate risk acute myeloid leukemia in first complete remission underwent ASCT from an HLA-matched sibling. Her initial induction was complicated by hypoxia, pulmonary infiltrates, and fevers despite broad-spectrum antibiotics. She was treated with voriconazole with clinical improvement and was presumed to have invasive fungal infection. Voriconazole was continued throughout her transplant course given persistent chest CT abnormalities.

**Figure 1:**
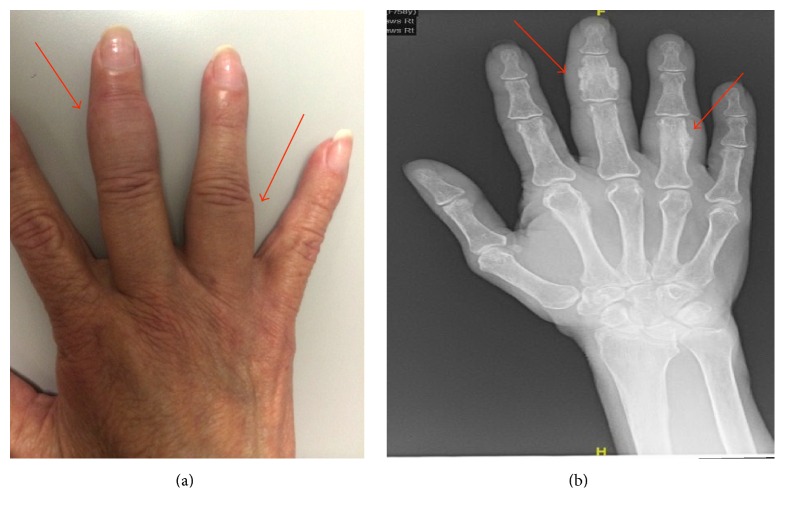
Examination findings and radiograph illustrating periostitis of the right hand. (a) Examination revealed swelling with tenderness of the third middle phalange as well as the less marked swelling of the fourth proximal phalange. (b) Radiograph showed multifocal periosteal reaction with associated soft tissue swelling.

At day 79 after ASCT, the patient complained of swelling in her left middle finger. Physical examination revealed discrete tender swelling of the middle phalanx on the third finger. This continued to worsen and one month later she was complaining of further swelling involving the fourth finger (Figure 1(a)). In addition, she reported fatigue and proximal muscle myalgia especially in her lower extremities. An X-ray of her hand (Figure 1(b)) reported multifocal periosteal reaction involving multiple bones of the right hand with associated soft tissue swelling. Laboratory testing showed elevated alkaline phosphatase (ALP) of 341 u/L (normal 40–125 u/L). Her liver function tests and kidney function were within normal limits. Erythrocyte sedimentation rate was mildly elevated at 31 mm/hr (normal 0–10 mm/hr). C reactive protein (CRP) and creatine kinase (CK) levels were normal. At that time, she was prescribed 1 milligram per kilogram per day of prednisone for musculoskeletal complaints associated with a presumed diagnosis of chronic graft-versus-host disease (cGVHD). However, she had only minor improvement in her symptoms after one week of treatment. An MRI of the hand was performed and showed exuberant disorganized periostitis involving the second, third, and fourth digits. It was suspected that the patient had voriconazole-induced periostitis. Of note, she had received voriconazole for 6 months with trough concentrations within therapeutic range. Voriconazole was discontinued and one week later the patient's symptoms began improving. Her ALP level improved from 341 u/L to 179 u/L and continued to improve over the next couple weeks. At day 175 after transplant, her symptoms had completely resolved.

## 3. Discussion

Voriconazole is a triazole antifungal that is indicated for the treatment of invasive aspergillosis. Common adverse effects include visual disturbances, hallucinations, QT prolongation, and hepatotoxicity. With prolonged use however, newly described adverse effects, including periostitis, alopecia, and development of skin cancers, have been noted [1]. Painful periostitis is a well-recognized, albeit uncommon, complication of prolonged voriconazole therapy [4–7]. The clinical features of voriconazole-induced periostitis are similar to skeletal fluorosis. As opposed to other azole antifungals, voriconazole is trifluorinated [8]. Therefore it is theorized that periostitis occurs due to high circulating levels of fluoride released during hepatic metabolism. The calculated daily fluoride intake at a standard dose of voriconazole may be as high as 62.6 mg which exceeds the fluoride toxicity threshold as defined by the World Health Organization 10-fold [8–10].

Most cases of voriconazole-induced periostitis have been reported in patients who have undergone solid organ transplantation [4–7]. Few cases in ASCT patients have been published [8, 11, 12]. Barajas et al. [12] have retrospectively analyzed 242 ASCT patients who received prolonged voriconazole treatment. Twenty-nine of 31 patients who experienced pain had elevated fluoride levels. These patients, however, did not have confirmed radiologic diagnosis of periostitis. Commonly described locations of involvement among both solid organ and ASCT patients are the clavicles, ribs, scapula, acetabulum, and hands [8–10, 12]. Patients typically complain of bone pain that is not alleviated by analgesics. Symptoms can occur within days of starting treatment but typically are seen after 3 to 6 months of therapy. Prompt discontinuation of the drug results in resolution of symptoms within a few weeks [10].

The clinical presentation and radiologic findings of patients who develop voriconazole-induced periostitis are nonspecific and insidious and may be confused with rheumatologic or endocrinologic disorders. In the setting of ASCT, symptoms may mimic cGVHD. Prior cases reported in the ASCT setting were also treated with corticosteroids for cGVHD, resulting in temporary symptom relief and a delay in discontinuing voriconazole [9–11]. In our case, there was a 3-month delay from time of symptom onset to voriconazole discontinuation due to a presumed diagnosis of cGVHD. Laboratory tests that help differentiate cGVHD from periostitis include ALP level, CRP, CK, and rheumatologic testing. Alkaline phosphatase levels are often elevated in periostitis but normalize within 1 to 2 months after discontinuation [4–7].

Given that long-term voriconazole therapy is increasingly common, clinicians managing ASCT patients should be aware of this complication. We recommend periodic measurement of ALP and fluoride levels in patients on prolonged voriconazole therapy. In patients on voriconazole who report nonspecific musculoskeletal complaints following ASCT, drug-induced periostitis should be included in the differential diagnosis along with cGVHD.
